# Impact of Cover Crops on the Soil Microbiome of Tree Crops

**DOI:** 10.3390/microorganisms8030328

**Published:** 2020-02-26

**Authors:** Antonio Castellano-Hinojosa, Sarah L. Strauss

**Affiliations:** Department of Soil and Water Sciences, Southwest Florida Research and Education Center, Institute of Food and Agricultural Sciences, University of Florida, Immokalee, FL 34142, USA; antonio.castella@ufl.edu

**Keywords:** microbial diversity, microbial abundance, intercropping, orchard, perennial cropping systems, soil health, nitrogen fixation, nitrification, denitrification, soil organic matter

## Abstract

Increased concerns associated with interactions between herbicides, inorganic fertilizers, soil nutrient availability, and plant phytotoxicity in perennial tree crop production systems have renewed interest in the use of cover crops in the inter-row middles or between trees as an alternative sustainable management strategy for these systems. Although interactions between the soil microbiome and cover crops have been examined for annual cropping systems, there are critical differences in management and growth in perennial cropping systems that can influence the soil microbiome and, therefore, the response to cover crops. Here, we discuss the importance of cover crops in tree cropping systems using multispecies cover crop mixtures and minimum tillage and no-tillage to not only enhance the soil microbiome but also carbon, nitrogen, and phosphorus cycling compared to monocropping, conventional tillage, and inorganic fertilization. We also identify potentially important taxa and research gaps that need to be addressed to facilitate assessments of the relationships between cover crops, soil microbes, and the health of tree crops. Additional evaluations of the interactions between the soil microbiome, cover crops, nutrient cycling, and tree performance will allow for more effective and sustainable management of perennial cropping systems.

## 1. Introduction

**Figure 1:**
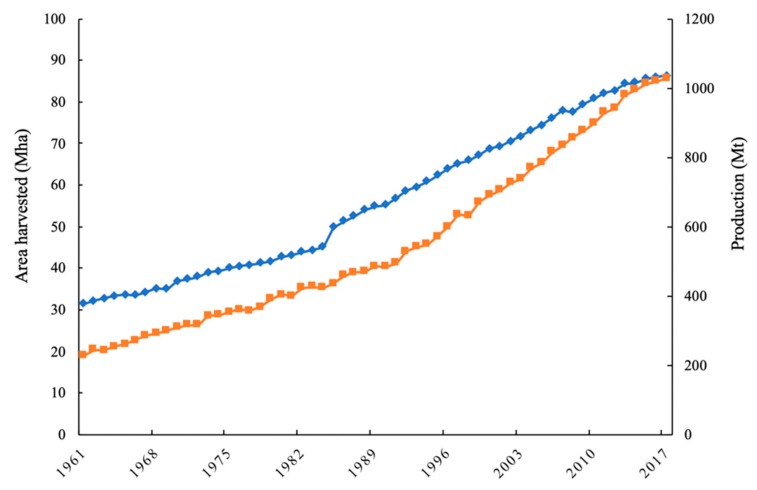
Total world area harvested (blue) and production (orange) of tree crops during the period 1961–2017 [1].

Perennial tree crops account for over 10% of global agriculture production according to the United Nations Food and Agriculture Organization (FAO). In the last 56 years, the global surface area covered by tree crops has increased to an approximate global harvested area of 86.3 Mha in 2017 [1] (Figure 1). In 2017, the most cultivated tree crops in the world were grapes, cashews, and mangoes, accounting for a global harvested area of 69.3, 59.8, and 56.8 Mha, respectively [1]. Management of these perennial systems can be intensive, and fertigation and foliar applications of inorganic fertilizers are frequently performed to satisfy the high nutrient demand of tree crops [2,3,4,5]. In addition, herbicides are often applied for row middle vegetation management, which may result in the development of herbicide resistance [6,7]. Concerns associated with interactions between herbicides, inorganic fertilizers, soil nutrient availability, and plant phytotoxicity have been raised for several perennial tree crop production systems [8,9,10,11], increasing interest in alternative management strategies for these systems.

Cover crops are a widely applied soil improvement and conservation technique in agriculture, particularly in annual cropping systems [12,13], and their implementation in tree cropping systems may provide the same benefits found in annual crops, including improved soil and root health; increased production; and reductions in costs due to decreases in fertilizers, irrigation, and herbicides [11,14]. Cover cropping can also improve soil carbon (C), nitrogen (N), and phosphorus (P) cycling [15,16,17], as well as increase soil microbial abundance and diversity [18] and suppress soilborne pests [19].

**Figure 2:**
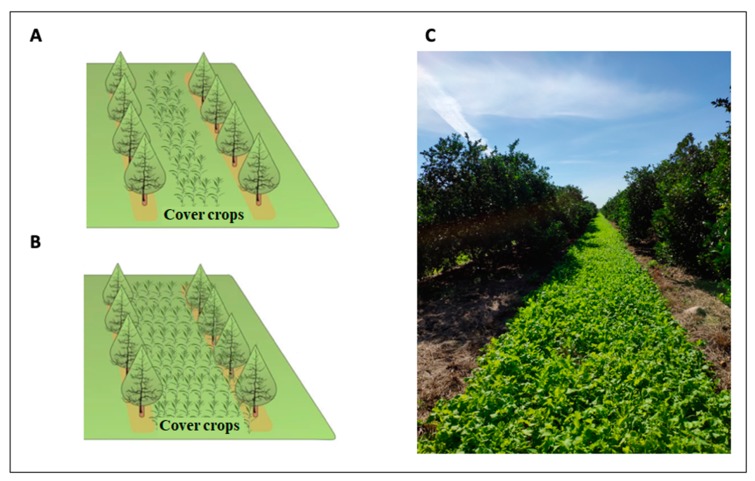
Cover crops can be planted in the inter-row middles (**A**) or between trees (**B**) in perennial systems. In warmer climates, such as in Florida for citrus, year-round cover crop planting is possible for perennial systems (**C**).

The majority of research on the benefits and impacts of cover crops to agroecosystems has focused on annual cash crops. In annual cropping systems, cover crops are primarily planted during the fallow season, often for only 3–5 months, when the cash crop is not in production [12,13]. The cover crop is then terminated and incorporated into the soil before planting the cash crop. However, as perennial crops often remain in production for over 20 years [20], there are critical differences in management and growth compared with annual cropping systems that can influence the soil microbiome and, therefore, the response to cover crops. In perennial systems, cover crops are planted in the inter-row middles or between trees (trunk-to-trunk, herein called “intercropping”; Figure 2); the latter is a less common practice due to potential competition for water between the tree and the cover crops when coverage is greater than 45% of the tree canopy [21]. Regardless of whether cover crops are planted in the inter-row or between trees, there is potential for both the tree and cover crops to share resources [12]. Unlike annual cropping systems, annual rotation and soil tillage are not present in perennial systems, which potentially allows for greater increases in soil organic matter (SOM) [11,22] and the development of more stable communities of beneficial microbial communities [23]. The timing of cover crop planting is another large difference between annual and perennial systems. In warmer climates, such as in Florida for citrus, year-round cover crop planting is possible for perennial systems, requiring multiple plantings and careful selection of cover crop species for each season. The limited space planted to cover crops in perennial compared with annual systems may also prolong the time needed to increase nutrient cycling [14]. However, in both perennial and annual systems, indirect effects on the soil microbial community through agricultural inputs (i.e., application of fertilizers, pesticides, and irrigation) may be similar.

The relationship between cover crops and the soil microbial community is integral to the influence of cover crops on the productivity of the cash crop and the benefit to the agroecosystem. Plants positively and negatively select for members of their phytobiome through the release of chemical signals into their environment [24,25] and establish a soil microbiome that can benefit plant growth [26,27]. Although interactions between plants and microbes have generally been studied with respect to individual microbes and/or specific crops [25,28,29,30], examinations of potentially important taxa associated with cover crops in perennial production systems are still lacking. Incorporating soil–plant–microbe interactions into production systems is an essential step for alternative and sustainable management strategies aimed at increasing the abundance and diversity of beneficial microbes that can play a fundamental role in nutrient cycling and ultimately cash crop production [31,32,33,34].

However, uncovering potential microbial drivers of the association between cover and tree crops in perennial systems is challenging due to the complexity of agroforestry systems and the limited number of trees species and cover crops examined. To date, only 20 studies have examined the interactions between belowground microbial communities associated with cover crops in tree cropping systems (Table 1). Differences in management between annual and perennial systems also require separate evaluation of the influence of cover crops on the soil microbiome. Therefore, the specific aims of this review are to describe (1) our current understanding of the effect of cover crops in perennial production systems on the abundance, diversity, and function of soil microbial communities; (2) the effect of cover crops on microbial communities of the N, C, and P biogeochemical cycles and their impact on nutrient availability; and (3) potentially important taxa associated with cover crops. Finally, we suggest future perspectives to facilitate assessments of the relationships between cover crops, soil microbes, and the health of tree crops.

## 2. Cover Crops Increase Soil Microbial Diversity

Cover crops can impact the soil microbiome by changing soil characteristics (e.g., pH, temperature, and soil water content) which are known to influence soil microbial communities [11,18,23]. In addition, cover crops offer additional organic substrates through the input of plant residues and rhizodeposition, which may impact soil microbial communities [55,56,57].

These changing soil conditions and increased organic inputs likely drive the increased microbial diversity found in soils of perennial systems planted with cover crops [11,14,58]. This increased soil microbial diversity is positively correlated with nearly all of the benefits of cover crops to production systems, including increased soil organic carbon (SOC) and total N (TN) contents and, ultimately, tree production [36,37,38,39,40,41,42,43,44,45,46,47,48,49,50,51,52,53,54] (Table 1). Changes can persist throughout the long-term management of a perennial system, as 10 and 22 years of planting a monoculture cover crop of *F. macrophylla* in rubber (*E. urophylla*) orchards increased bacterial gene abundance and diversity down to a depth of 60 cm within the soil profile compared with the no-cover-crop control treatment [35].

Planting multispecies cover crop mixtures, which is the combination of at least two legume or nonlegume species [59], may provide additional benefits by not only increasing microbial diversity but also the abundance of beneficial soil microbes, such as rhizosphere bacteria (*Azospirillum* sp., *Azotobacter* sp., *Bacillus* sp., and *Pseudomonas* sp.) and mycorrhizal fungi (*Acaulospora morrowiae*, *Archaeospora trappei*, *Gigaspora gigantea*, and *Scutellospora calospora*), compared with monocultures [60,61,62]. The positive effect of cover crop mixtures is not altogether surprising given the positive relationship between plant biodiversity, the higher availability and diversity of root exudates, and soil microbial diversity [63,64,65,66].

Tillage in the row middles is common for some perennial crops, such as almonds, olives, citrus, and grapevines [11,67], and is generally performed to remove weeds to avoid water and nutrient competition, resulting in bare soil between tree rows [68,69]. Adopting no-tillage in combination with cover crops has been identified as a reliable management practice in tree cropping systems [70,71] that increases soil C sequestration and reduces N fertilization inputs compared with conventional tillage (down to a depth of 15–20 cm), primarily due to the beneficial effects on soil microbial diversity [11,14,18,23]. For example, intercropping olive (*O. europea*) with grasses increased bacterial diversity in no-tillage systems compared with conventional treatments, and changes in microbial diversity were positively correlated with improved olive yield [49]. Further, intercropping *Lolium multiflorum* and *Medicago* sp. cover crops in an apricot (*P. armeniaca*) orchard increased the diversity of total bacteria, actinomycetes, proteolytic bacteria, *Pseudomonas* sp., *Azotobacter* sp., and ammonifying bacteria under no-tillage compared with tillage treatments and was correlated with increased soil fertility [36].

Cover crop termination methods can also impact microbial diversity in perennial systems. The most common cover crop termination strategies are employed chemically by application of herbicides [72], naturally by frost, and mechanically by rolling the cover crops with a roller crimper [14,73]. The termination of cover crops with herbicides reduced the abundance and diversity of bacterial communities compared with a nonherbicide treatment in olive orchards [53]. However, while herbicides reduced the abundance and diversity, they had little impact on the microbial activity of six enzymes involved in C, P, and N cycling, and herbicide-terminated cover crop treatments still increased soil nutrient availability [53]. This suggests that cover crops may mitigate the negative impact of herbicides on soil microbial diversity and activity, therefore still improving nutrient cycling in tree cropping systems.

The assessment of cover crop impacts on the soil fungal communities in perennial systems is less common than bacterial assessments, and five published studies have shown increases in the diversity of fungal communities when crops were used in tree cropping systems (Table 1). Intercropping apple (*M. domestica*) with grasses significantly increased arbuscular mycorrhizal fungal (AMF) richness with respect to integrated treatments (cover crops with inorganic N fertilizer) [47,49]. Specifically, the relative abundance of members of the fungal genera *Glomus*, *Paraglomus*, *Claroideoglomus*, *Sclerocystis*, and *Rhizoglomus* significantly increased in the cover crop treatments and were linked to increased crop productivity.

### 2.1. Carbon Cycle

Cover crops may enhance soil properties and tree yield via the integrated adjustment of N and P cycling and SOM turnover [35,42]. One of the primary methods for increasing SOM is through the degradation of cover crop residues (Table 1). For example, in a rubber orchard, *F. macrophylla* as a cover crop increased the relative abundance of copiotrophic members of the phyla Actinobacteria, Bacteroidetes, and Proteobacteria, which were positively correlated with an increase in SOM degradation [35]. Planting *F. arundinacea* also increased the SOM content by 7% over 7 years of applications in organic apple orchards [46]. The relative abundance of members of the phyla Firmicutes (*Bacillus*), Proteobacteria (Rhizobiales), Acidobacteria, and Actinobacteria was also positively correlated with the decomposition of soil organic materials such as cellulose and chitin when wheat was used as a cover crop in a walnut orchard [42]. Fungal taxa, key for plant residue degradation, increased in olive orchards intercropped with grass cover crops compared with no-cover-crop treatments and were correlated with increased SOM [50,51,53].

The abundance of SOM metabolism-related genes and soil enzymes also increased with cover crops. In an apple orchard [43], 41% of the genes related to the degradation of cellulose, hemicellulose, and cello-oligosaccharides were significantly more abundant in the cover crop treatments due to the input of cover crop residues. In addition, the relative abundance of 22 selected taxa of the Firmicutes and Bacteroidetes phyla related to the breakdown of plant polymers such as cellulose, hemicellulose, and cello-oligosaccharides significantly increased in apple orchard soils planted with the cover crop *V. villosa* compared with a non-cover-crop control and were correlated with increases in SOM content [44,45]. No-tillage management practices together with the use of grass cover crops increased soil enzymatic activities with a concomitant increase in SOM content in Mediterranean olive orchards [51,53] when compared with conventional tillage with N fertilization treatments.

### 2.2. Nitrogen Cycle

One of the key potential benefits to crop production from cover crops, both in perennial and annual systems, is the input of N and the influence on soil N availability. As with annual crops [74], N-cycling microorganisms are crucial for the sustainability of a tree cropping production system as they are linked to N cycling availability and, ultimately, soil and tree health. Accordingly, in this section, we highlight the effect of cover crops on N-cycling microbial communities with an emphasis on three major routes of the N cycle: N_2_-fixation, nitrification, and denitrification.

#### 2.2.1. N_2_-Fixation

The use of legume cover crops in tree cropping systems is expected to provide N to the soil through the process of N_2_-fixation, during which atmospheric N (N_2_) is reduced to ammonium (NH_4_^+^) in root nodules of leguminous plants [75]. Biological N_2_-fixation is catalyzed by nitrogenase, a complex enzyme that has two components: a heterotetrameric core encoded by *nifD* and *nifK* genes, and a dinitrogenase reductase subunit encoded by *nifH*. The *nifH* gene is the biomarker most widely used to study the abundance and diversity of N_2_-fixing bacteria [76].

The relationship between the abundance of the *nifH* gene and legume cover crops in perennial systems is not clear or well studied (Table 1), though there are positive correlations between *nifH* gene abundance and N availability. When comparing a legume cover crop (*V. villosa*) to a standard inorganic N fertilizer application in a vineyard orchard for 10 years, *nifH* abundance was significantly greater under the legume [41]. This is not surprising, as *nifH* abundance and N_2_-fixation are often limited in soils with high inorganic N concentrations [77]. By contrast, intercropping *Coronilla varia* in an apple orchard for 9 years significantly increased the content of soil N but did not alter the abundance of the *nifD* and *nifK* genes compared to the non-cover-crop treatment [43]. While Pereg et al. [41] used a qPCR approach and the *nifH* gene as a molecular marker to study the abundance of N_2_-fixing bacteria, Zheng et al. [43] estimated the size of the N_2_-fixing community by amplicon sequencing, using the *nifD* and *nifK* genes as biomarkers. It is possible that the use of different molecular approaches and targeted genes to estimate the size of the N_2_-fixing community explains the different effects of legume cover crops on the N_2_-fixation gene abundances between these two studies. For example, primers for quantification of N-cycling genes do not always cover all species with the target gene [78], and estimation of microbial gene abundance appears to be more accurate using metagenome sequencing [79].

While rhizobia associated with legumes are the most common method for providing additional N to soils through cover crops, free-living N_2_-fixation could potentially contribute N to perennial systems. Free-living N_2_-fixation, defined as N_2_-fixation occurring without a formal plant–microbe symbiosis, is an important process distinct from symbiotic N_2_-fixation in the rhizosphere of legume cover crops [80]. Indeed, free-living N_2_-fixation is a ubiquitous process in terrestrial systems and can provide significant inputs of N equal to or greater than symbiotic N_2_-fixation [80,81], even in perennial crops. For example, regardless of the presence of legume cover crops, Morales et al. [82] found similar abundances of the *nifH* gene between perennial and annual systems, indicating the importance of free-living N_2_-fixation in tree cropping systems.

#### 2.2.2. Nitrification

Ammonia-oxidizing bacteria (AOB) and ammonia-oxidizing archaea (AOA) coexist in soils and perform the first (and rate-limiting) step in nitrification: the oxidization of NH_4_^+^ to nitrate (NO_3_^−^). However, the relative distribution of AOB and AOA and community composition vary depending on the environmental conditions [83,84], including soil pH [85] and forms of soil N [86]. Both phyla encode for the enzyme ammonia mono-oxygenase (*amo*), which can be environmentally traced by examining the gene coding for the alpha subunit of the enzyme (*amoA*).

Legume cover crops [35,36,43] can increase the abundance of nitrification genes in perennial systems (Table 1). In legumes, the abundance of AOB and AOA appears to be positively related to increased N availability. For example, after 9 years of intercropping *C. varia* in an apple orchard, the total abundance of *amoA* AOB and *amoA* AOA genes was significantly greater compared with the non-cover-crop treatment and positively correlated with soil concentrations of NH_4_^+^ and NO_3_^−^ [43]. Increases in AOB and AOA in nonlegume cover crops have been reported in annual systems, likely related to the increases in organic inputs from cover crop residues [87,88,89]. However, to date, no studies have evaluated the effect of nonlegume cover crops on the abundance of nitrification genes in perennial systems.

There is limited information regarding the impact of cover crops on the nitrifying microbial community in perennial systems. In general, intercropping grasses in an olive orchard for 15 years did not affect the composition of the nitrifying community in the olive rhizosphere [54]. When cover crops were controlled by mowing or herbicides instead of by grazing, the abundance of AOB was significantly increased, an effect that was associated with increased soil organic C in the mowing and herbicide treatments possibly due to a greater presence of cover crop residues that were subjected to mineralization [54]. However, these studies only examined nonlegume cover crops, and the impact of legume cover crops remains unexplored.

#### 2.2.3. Denitrification

Denitrification is a step-by-step pathway that ultimately reduces NO_3_^−^ to N_2_ and comprises the enzymes nitrate, nitrite, nitric oxide, and nitrous oxide reductases, encoded by the *napA*/*narG*, *nirK*/*nirS*, *norB*, and *nosZ* structural genes, respectively [90]. Denitrification gene abundances have increased when planting cover crops in tree cropping systems [41,43,46] (Table 1). In most cases, such increases are directly related to the presence of organic amendments such as cover crop residues (e.g., pruning and mulch), which are sources of organic C, one of the most important factors influencing denitrification [91]. In a vineyard production system, the combined use of grapevine pruning and *V. villosa* as a cover crop had similar abundances of *nirK*, *nirS*, and *nosZ* genes as an organic treatment (pruning with manure), but abundances significantly increased when compared with an inorganic N fertilizer treatment [41]. Cover crop influences on biological and physical soil properties, such as available N, soil microbial biomass, enzymatic activity, and aggregate stability, are also related to the abundance of denitrification genes. For example, the increase in the abundance and diversity of *nirK*-type denitrifiers in an apple orchard with cover crop treatments of *F. arundinacea* was positively correlated with the content of dissolved organic nitrogen (DON) in the soil [46]. The high C:N ratio of intercropping *V. villosa* in an apple orchard was correlated with the increased abundance of *nirB* and *nirD* genes and SOM content compared with the non-cover-crop control treatment [43].

### 2.3. Phosphorus Cycle

Despite soil P existing in multiple chemical forms, including inorganic P (Pi) and organic P (Po), it is often unavailable to plants and microbes due to its slow diffusion and high fixation to soil particles [92,93]. Soil available P can be released either by mineralization of Po [94] or directly by soil microbes [95].

Three homologous genes encoding alkaline phosphomonoesterases (APase) in prokaryotes have been identified and are the most widely used biomarkers to study the ecology and evolution of P-solubilizing bacteria: *phoD* [96], *phoA* [97], and *phoX* [98]. In addition, the *pqqC* gene that encodes the pyrroloquinoline–quinone synthase C [99] and the E3.1.3.8 gene that encodes the production of phytate [100] can be used as a molecular marker to study the diversity of P-solubilizing microbes.

Inoculation of cover crops with selected rhizobia and AMF strains can effectively improve the availability of P in soils, which can promote the growth of cash crops [101]. In a guava (*P. guajava*) orchard, the combined application of cover crops (*Paspalum natatu* and *Stylosanthes guianensis*) and symbiotic microbes (rhizobia and/or AMF) significantly increased the content of organic P and phosphorus-related enzymatic activities and the abundance and diversity of APase-harboring bacterial communities compared with cover cropping alone [37]. In addition, utilization of the cover crops resulted in a significant increase in the number of spores of AMF, which may play a crucial role in the absorption and transportation of organic P [102]. However, cover crops alone might be enough to enhance P-solubilizing communities, as the relative abundances of the *phoA*, *pqqC*, and E3.1.3.8 genes significantly increased in an apple orchard intercropped with *V. villosa* for 9 years and were positively correlated to the soil P content compared with the non-cover-crops [43]. Microbial network analysis showed that the members Proteobacteria, Acidobacteria, and Actinobacteria were the main drives of P reactions in the cover crop treatments [43].

## 3. Linking Microbial Diversity to Function in Perennial Systems

While examining the impact of cover crops on soil microbial diversity provides an assessment of the overall change to the microbial community composition, it is difficult to link these composition changes to functional changes in the soil microbiome and, therefore, the direct influence of cover crops on factors influencing the cash crop. Shotgun metagenomic sequencing is currently the most common tool for determining the functional microbial composition of soils [103]. However, tools such as PICRUSt2 [104], Tax4Fun2 [105], and FAPROTAX [106] can predict functional profiles and functional redundancy of prokaryotic communities from 16S rRNA gene sequences. Together, these tools help identify potentially important taxa responsible for functional differences between microbiomes, a promising approach towards manipulating microbiomes to increase soil health and plant fertility. In addition, microbial network analysis based on co-occurrence patterns is being employed to study the relationships between different taxa and may help to identify interactions between microbial communities, habitat preference, or keystone species that exert larger effects on ecosystem processes (i.e., nutrient cycling) that could guide more focused experimental settings [107,108,109].

There are limited studies on cover crops in perennial systems utilizing these methods, but cover crops appear to alter the structure and function of the soil microbiome network, increasing intertaxa associations in soils which may result in increases in the number of metabolic pathways associated with nutrient cycling and the abundance of beneficial microbes. Bacterial communities were more connected in cover crop than non-cover-crop treatments in apple orchards intercropped with crown vetch (*Coronilla varia*) and resulted in an increase in the number of links for plant degradation, as well as N and P reactions [44,45]. Members of the phylum Firmicutes, the order Clostridiales, and the families Ruminococcaceae and Lachnospiraceae were positively correlated not only to the content of SOC and TN but also to microbial community functions related to cover crop residue degradation [44,45]. The cover crop alone had stronger positive effects on the connections between taxa than the cover crop with N fertilizer treatment, suggesting that cover crops greatly influenced the composition of the soil microbial community compared with inorganic fertilizers. In addition, Capó-Bauçà et al. [38] found that the use of no-tillage and cover crops (*Medicago polymorpha*, *Avena* sp., *Cynodon dactylon*, and *Hordeum murinum*) in vineyard systems increased the microbial functional diversity, as the microbial community from soils under green cover was able to degrade 10 more substances than from cover crop and tillage soils.

## 4. Potentially Important Microbes Associated with Cover Crops in Perennial Systems

**Figure 3:**
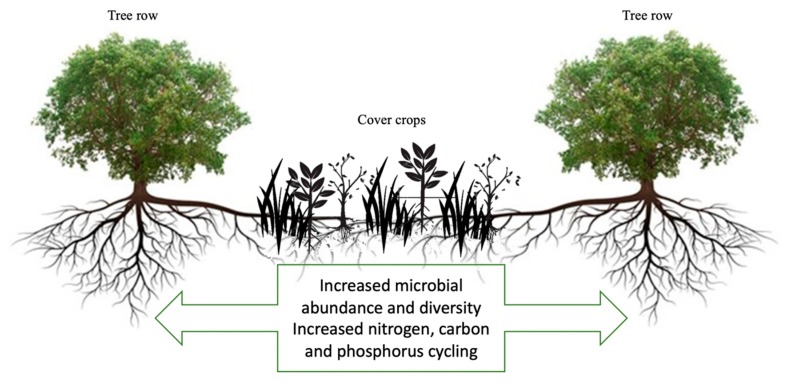
Illustration of the impacts of cover crops on tree crops.

As mentioned throughout this review, utilization of cover crops in the inter-row between trees affects the abundance, diversity, and function of soil microbial communities, with potential beneficial effects on soil and tree health (Figure 3). Recent studies on the impact of different plants on the soil microbiome have identified highly connected taxa that individually or in a guild confer particular functions to their host, irrespective of their abundance [110]. These potentially important taxa are often pertinent to the major shifts in the whole community structure and their identification can be a reliable strategy to gain fundamental understanding of both plant–microbe and microbe–microbe coevolution [111]. As shown in this review, it is reasonable to suppose that cover crops exert important influences on bacterial and fungal co-occurrence networks. However, while a recent meta-analysis found cover cropping increased soil microbial abundance, activity, and diversity in annuals [18], considerations for potentially important taxa associated with cover crops in annual and perennial systems were not discussed.

From the set of published studies on the effect of cover crops on the soil microbiome in tree cropping systems (Table 1), we selected bacterial, archaeal, and fungal taxa from each study that had a significant and positive correlation to at least one of the following abiotic parameters that are commonly used as indicators of soil quality [112]: content of NH_4_^+^, NO_3_^−^, TN, and SOM; decomposition of complex polymers; and tree production.

Bacteria are the most studied potential microbial indicators for soil quality [33,113]. Across multiple studies (Table 1), regardless of the use of nonlegume or legume cover crops, the relative abundance of members of the phyla Acidobacteria, Actinobacteria, Bacteroidetes, Firmicutes (*Bacillus*), Proteobacteria (*Azotobacter*, *Nitrobacter*, *Pseudomonas*, and Rhizobiales), Tenericutes, and Verrucomicrobia was positively related to a general improvement in N availability and SOM content in tree cropping systems [35,36,37,38,39,40,43,44,45]. Members of the abovementioned bacterial phyla are the most common drivers of soil health [33,113]. When looking at the genera level, the taxa associated with improved nutrient cycling in tree cropping systems differ across studies and seem to be both tree crop and cover crop specific.

The relative importance of archaeal taxa in tree cropping systems is largely unknown, but *Nitrososphaera* were the predominant archaeal taxa in the rhizosphere of olive orchards intercropped with unspecified grasses and positively correlated to the content of organic N and exchangeable potassium [54]. *Nitrososphaera* are the most represented AOA taxa in soils [114,115], and increases in their relative abundance are linked to increased NH_4_^+^ content [116,117], suggesting that these microorganisms could be used as biomarkers of soil health in tree cropping systems.

Despite the importance and role of fungal communities in soil health [118], only five studies have examined the diversity and function of these microorganisms in perennial cropping systems. The fungal genera *Acremonium*, *Alternaria*, *Armillaria*, *Aspergillus*, *Cladosporium*, *Cylindrocarpon*, *Microdochium*, *Penicillium*, *Phaeoacremonium*, *Phialophora*, and *Rosellini* were positively related to increased SOM content and decomposition of complex polymers in olive orchards intercropped with grass cover crops [50,51,53] and their beneficial roles in soil health have been previously described in agricultural soils [52]. Whether the presence of the abovementioned fungal taxa is indicative of improved soil health, regardless of the cover crop and tree crop used, remains unexplored.

## 5. Conclusions and Future Perspectives

Increased interest in improving the sustainability of agriculture and availability of soil nutrients has led to renewed attention to cover cropping as an agricultural practice with benefits for growers and the environment. As with annual production systems, cover crops in tree cropping systems increase microbial abundance and diversity with concomitant positive effects on C, N, and P cycling. Particularly, intercropping with multispecies cover crop mixtures and minimum tillage and no-tillage not only enhances the soil microbiome but also SOM and N contents compared with monocropping, conventional tillage, and inorganic fertilization. Cover crop residues can also be a suitable strategy to increase the abundance of SOM-related genes, promoting plant degradation and, ultimately, increases in the content of SOM.

However, many important gaps regarding the effect of cover crops on the soil microbiome in tree cropping systems exist. For example, the extent to which different combinations of cover crops (e.g., different mixtures of nonlegume and legume cover crops) impact nutrient cycling and the soil microbiome is largely unknown and seems to be both cover crop and tree crop specific. The majority of existing cover crop studies have focused on cereal and/or grain crops, and while there are examples of significant impacts of cover crop use with tree crops, less is known about the varieties, timing, and contribution of cover crops to perennial agroecosystems. Further, due to differences in management between annual and perennial systems, the effect of cover crops on the tree rhizosphere microbiome is largely unknown as well as that of multiple plantings of cover crop species. The limited space planted to cover crops in perennial compared with annual systems may also prolong the time needed to increase nutrient cycling and provoke a spatial variation in the soil microbiome between the row middle and the tree row (Figure 3).

Measuring changes of the soil microbiome associated with cover crops in tree cropping systems and linking them to ecosystem functions through the prediction of functional profiles and the use of microbial network analyses is a promising strategy to identify potentially important taxa in perennial systems. Although knowledge of potentially important taxa associated with cover crops may allow for manipulation of the soil microbiome, the extent to which planting different combinations of cover crops could be used to select beneficial microbes with particular roles in nutrient cycling and, ultimately, tree production is largely unknown. Future research should also include methodical isolation of potentially important microbiota, not only for in situ testing for their use as microbial inoculants but also to verify their role as potentially important taxa. Together, this information will provide a more detailed understanding of the molecular mechanisms used by plants to interact with the soil microbiome and will allow for the development of more sustainable agricultural production for perennial cropping systems.

## Figures and Tables

**Table 1:** Summary of studies comparing the effect of cover crop treatments on soil microbial communities in woody perennial systems.

Cover Crop Type	Tree Crop	Microbial Determination Method *	Reference
*Flemingia macrophylla*	*Eucalyptus urophylla*	qPCR (16S rRNA gene) and Illumina sequencing (16S rRNA gene)	[35]
*Lolium multiflorum* and *Medicago* sp.	*Prunus armeniaca*	Cultivable microorganisms (actinomycetes, ammonifying and proteolytic bacteria, *Azotobacter* sp., *Pseudomonas* sp.)	[36]
*Paspalum natatu* and *Stylosanthes guianensis*	*Psidium guajava*	DGGE (16S rRNA)	[37]
*Erodium chium*, *Medicago polymorpha*, *Avena* sp., *Cynodon dactylon*, and *Hordeum murinum*	*Vitis vinifera*	Biolog EcoPlate and number of mycorrhizal spores	[38]
Unspecified noncereal grasses, *Pisum sativum*, *Phaseolus* sp., *Trifolium* sp., *Vicia* sp., and *Sinapsis* sp.	*V. vinifera*	Illumina sequencing (16S rRNA gene)	[39,40]
*Vicia villosa*	*V. vinifera*	qPCR (16SB, *amoA* AOA, *amoA* AOB, *nifH*, *nirK*, *nirS*, *nosZ*)	[41]
*Triticum aestivum*	*Juglans regi*	Illumina sequencing (16S rRNA gene)	[42]
*Coronilla varia*	*Malus pumila*	Illumina sequencing (16S rRNA gene and ITS of fungi), metagenome sequencing (N, C, and P cycling genes), Tax4Fun, and PICRUSt	[43,44,45]
*Festuca arundinacea*	*Malus domestica*	DGGE (*nirK* gene)	[46]
Unspecified grasses	*M. domestica*	DGGE (16S rRNA gene)	[47]
*Symphytum* × *uplandicum*, *Allium tuberosum*, *Rumex acetosa*, *Lupinus perennis*, *Trifolium repens*, *Mentha* × *piperita*, *Monarda fistulosa*, *Allium proliferum*, and *Caragana arborescens* (*F. arundinacea*, *Festuca rubra*, and *Lolium perenne)*	*M. domestica*	qPCR (16S rRNA, 18S rRNA, and ITS genes)	[48]
Unspecified grasses	*Olea europaea*	Biolog Ecoplate, DGGE (16S rRNA and 18S rRNA genes), and cultivable microorganisms (actinomycetes, ammonifying and proteolytic bacteria, *Azotobacter* sp., *Streptomyces* sp.)	[49,50]
Unspecified grasses	*O. europaea*	T-RFLP (arbuscular mycorrhizal of fungi), Biolog EcoPlate, and pyrosequencing (arbuscular mycorrhizal of fungi)	[51]
Unspecified grasses	*O. europaea*	Pyrosequencing (16S rRNA gene)	[52]
Unspecified grasses	*O. europaea*	DGGE (16S rRNA gene) and qPCR (16S rRNA)	[53]
Unspecified grasses	*O. europaea*	qPCR (*amoA* AOB and *amoA* AOA genes), pyrosequencing (*amoA* AOB, *amoA* AOA, and nitrite-oxidizing bacteria)	[54]

* Denaturing gel electrophoresis (DGGE); terminal fragment polymorphism (T-RFLP); quantitative PCR (qPCR). Total bacterial (16S rRNA), fungal (18 rRNA; internal transcribed spacer, ITS); N_2_-fixing (*nifH*), nitrification (*amoA*), and denitrification (*nirK*, *nirS*, *nosZ*) genes.

## References

[1] United Nations Food and Agriculture Organization (FAO). http://www.fao.org/faostat/en/#data/QC.

[2] Improving the Nutrient Efficiency of Tree Crops. http://www.ucanr.org/sites/nm/files/76737.pdf.

[3] Fertilization of Perennial Tree Crops: Timing is Everything!. https://ucanr.edu/sites/nm/files/76732.pdf.

[4] Mattos D.J., Kadyampakeni D.M., Quiñones A., Marcelli R., Morgan K.T., Quagiio J.A., Talon M., Caruso M., Gmitter F.G. (2020). Soil and nutrition interactions. The Genus Citrus.

[5] Kadyampakeni D.M., Morgan K.T., Nkedi-Kizza P., Kasozi G.N. (2015). Nutrient management options for Florida citrus: A review of NPK application and analytical methods. J. Plant. Nutr..

[6] Singh M., Ramirez A.H.M., Jhala A.J., Malik M. (2012). Weed Control Efficacy and Citrus Response to Flazasulfuron Applied Alone or in Combination with Other Herbicides. Am. J. Plant Sci..

[7] Osipitan O.A., Dille J.A., Assefa Y., Knezevic S.Z. (2018). Cover crop for early season weed suppression in crops: Systematic review and meta-analysis. Agron. J..

[8] Huber D.M., Graham R.D., Rengel Z. (1999). The Role of Nutrition in Crop Resistance and Tolerance to Disease. Mineral Nutrition of Crops Fundamental Mechanisms and Implications.

[9] Johal G.S., Huber D.M. (2009). Glyphosate effects on diseases of plants. Eur. J. Agron..

[10] Kanissery R., Gairhe B., Kadyampakeni D., Batuman O., Alferez F. (2019). Glyphosate: Its Environmental Persistence and Impact on Crop Health and Nutrition. Plants.

[11] Morugán-Coronado A., Linares C., Gómez-López M.D., Faz Á., Zornoza R. (2020). The impact of intercropping, tillage and fertilizer type on soil and crop yield in fruit orchards under Mediterranean conditions: A meta-analysis of field studies. Agric. Syst..

[12] Schipanski M.E., Barbercheck M., Douglas M.R., Finney D.M., Haider K., Kaye J.P., Kemanian A.R., Mortensen D.A., Ryan M.R., Tooker J. (2014). A framework for evaluating ecosystem services provided by cover crops in agroecosystems. Agric. Syst..

[13] Kaye J.P., Quemada M. (2017). Using cover crops to mitigate and adapt to climate change. A review. Agron. Sustain. Dev..

[14] Vukicevich E., Lowery T., Bowen P., Úrbez-Torres J.R., Hart M. (2016). Cover crops to increase soil microbial diversity and mitigate decline in perennial agriculture. A review. Agron. Sustain. Dev..

[15] White C., Holmes H., Morris N. (2016). A review of the benefits, optimal crop management practices and knowledge gaps associated with different cover crop species. AHDB Cereal. Oilseeds. While.

[16] Almagro M., de Vente J., Boix-Fayos C., García-Franco N., Melgares de Aguilar J., González D., Solé-Benet A., Martínez-Mena M. (2016). Sustainable land management practices as providers of several ecosystem services under rainfed Mediterranean agroecosystems. Mitig. Adapt. Strateg. Glob. Chang..

[17] Wolff M.W., Alsina M.M., Stockert C.M., Khalsa S.D.S., Smart D.R. (2018). Minimum tillage of a cover crop lowers net GWP and sequesters soil carbon in a California vineyard. Soil Tillage Res..

[18] Kim N., Zabaloy M.C., Guan K., Villamil M.B. (2020). Do cover crops benefit soil microbiome? A meta-analysis of current research. Soil Biol. Biochem..

[19] Van Elsas J.D., Garbeva P., Salles J. (2002). Effects of agronomical measures on the microbial diversity of soils as related to the suppression of soil-borne plant pathogens. Biodegradation.

[20] Glover J.D., Reganold J.P., Bell L.W., Borevitz J., Brummer E.C., Buckler E.S., Cox C.M., Cox T.S., Crews T.E., Culman S.W. (2010). Increased food and ecosystem security via perennial grains. Science.

[21] Cao S., Lu C., Yue H. (2017). Optimal Tree Canopy Cover during Ecological Restoration: A Case Study of Possible Ecological Thresholds in Changting, China. Bioscience.

[22] Crews T., Rumsey B. (2017). What Agriculture Can Learn from Native Ecosystems in Building Soil Organic Matter: A Review. Sustainability.

[23] Mercado-Blanco J., Abrantes I., Caracciolo A.B., Bevivino A., Ciancio A., Grenni P., Hrynkiewicz K., Kredics L., Proença D.N. (2018). Belowground microbiota and the health of tree crops. Front. Microbiol..

[24] Sasse J., Martinoia E., Northen T. (2018). Feed Your Friends: Do Plant Exudates Shape the Root Microbiome?. Trends Plant Sci..

[25] Jones P., Garcia B.J., Furches A., Tuskan G.A., Jacobson D. (2019). Plant host-associated mechanisms for microbial selection. Front. Plant Sci..

[26] Ojuederie O.B., Babalola O.O. (2017). Microbial and plant-assisted bioremediation of heavy metal polluted environments: A review. Int. J. Environ. Res. Public Health.

[27] Syed Ab Rahman S.F., Singh E., Pieterse C.M.J., Schenk P.M. (2018). Emerging microbial biocontrol strategies for plant pathogens. Plant Sci..

[28] Van der Heijden M.G.A., Hartmann M. (2016). Networking in the Plant Microbiome. PLoS Biol..

[29] Banerjee S., Schlaeppi K., van der Heijden M.G.A. (2018). Keystone taxa as drivers of microbiome structure and functioning. Nat. Rev. Microbiol..

[30] Banerjee S., Walder F., Büchi L., Meyer M., Held A.Y., Gattinger A., Keller T., Charles R., van der Heijden M.G.A. (2019). Agricultural intensification reduces microbial network complexity and the abundance of keystone taxa in roots. ISME J..

[31] Macdonald C., Singh B. (2013). Harnessing plant-microbe interactions for enhancing farm productivity. Bioengineered.

[32] Jacoby R., Peukert M., Succurro A., Koprivova A., Kopriva S. (2017). The role of soil microorganisms in plant mineral nutrition—current knowledge and future directions. Front. Plant Sci..

[33] Trivedi P., Delgado-Baquerizo M., Anderson I.C., Singh B.K. (2016). Response of soil properties and microbial communities to agriculture: Implications for primary productivity and soil health indicators. Front. Plant Sci..

[34] Compant S., Samad A., Faist H., Sessitsch A. (2019). A review on the plant microbiome: Ecology, functions, and emerging trends in microbial application. J. Adv. Res..

[35] Liu C., Jin Y., Hu Y., Tang J., Xiong Q., Xu M., Bibi F., Beng K.C. (2019). Drivers of soil bacterial community structure and diversity in tropical agroforestry systems. Agric. Ecosyst. Environ..

[36] Casacchia T., Briccoli Bati C., Sofo A., Dichio B., Motta F., Xiloyannis C. (2010). Long-term consequences of tillage, organic amendments, residue management and localized irrigation on selected soil micro-flora groups in a Mediterranean apricot orchard. Acta Hortic..

[37] Cui H., Zhou Y., Gu Z., Zhu H., Fu S., Yao Q. (2015). The combined effects of cover crops and symbiotic microbes on phosphatase gene and organic phosphorus hydrolysis in subtropical orchard soils. Soil Biol. Biochem..

[38] Capó-Bauçà S., Marqués A., Llopis-Vidal N., Bota J., Baraza E. (2019). Long-term establishment of natural green cover provides agroecosystem services by improving soil quality in a Mediterranean vineyard. Ecol. Eng..

[39] Burns K.N., Kluepfel D.A., Strauss S.L., Bokulich N.A., Cantu D., Steenwerth K.L. (2015). Vineyard soil bacterial diversity and composition revealed by 16S rRNA genes: Differentiation by geographic features. Soil Biol. Biochem..

[40] Burns K.N., Bokulich N.A., Cantu D., Greenhut R.F., Kluepfel D.A., O’Geen A.T., Strauss S.L., Steenwerth K.L. (2016). Vineyard soil bacterial diversity and composition revealed by 16S rRNA genes: Differentiation by vineyard management. Soil Biol. Biochem..

[41] Pereg L., Morugán-Coronado A., McMillan M., García-Orenes F. (2018). Restoration of nitrogen cycling community in grapevine soil by a decade of organic fertilization. Soil Tillage Res..

[42] Gao P., Zheng X., Wang L., Liu B., Zhang S. (2019). Changes in the soil bacterial community in a chronosequence of temperate walnut-based intercropping systems. Forests.

[43] Zheng W., Zhao Z., Lv F., Wang R., Gong Q., Zhai B., Wang Z., Zhao Z., Li Z. (2019). Metagenomic exploration of the interactions between N and P cycling and SOM turnover in an apple orchard with a cover crop fertilized for 9 years. Biol. Fertil. Soils.

[44] Zheng W., Gong Q., Zhao Z., Liu J., Zhai B., Wang Z., Li Z. (2018). Changes in the soil bacterial community structure and enzyme activities after intercrop mulch with cover crop for eight years in an orchard. Eur. J. Soil Biol..

[45] Zheng W., Zhao Z., Gong Q., Zhai B., Li Z. (2018). Effects of cover crop in an apple orchard on microbial community composition, networks, and potential genes involved with degradation of crop residues in soil. Biol. Fertil. Soils.

[46] Jones J., Savin M.C., Rom C.R., Gbur E. (2017). Denitrifier community response to seven years of ground cover and nutrient management in an organic fruit tree orchard soil. Appl. Soil Ecol..

[47] Turrini A., Agnolucci M., Palla M., Tomé E., Tagliavini M., Scandellari F., Giovannetti M. (2017). Species diversity and community composition of native arbuscular mycorrhizal fungi in apple roots are affected by site and orchard management. Appl. Soil Ecol..

[48] Wartman P.C., Dunfield K.E., Khosla K., Loucks C., Van Acker R.C., Martin R.C. (2017). The establishment of apple orchards as temperate forest garden systems and their impact on indigenous bacterial and fungal population abundance in Southern Ontario, Canada. Renew. Agric. Food Syst..

[49] Sofo A., Palese A.M., Casacchia T., Celano G., Ricciuti P., Curci M., Crecchio C., Xiloyannis C. (2010). Genetic, functional, and metabolic responses of soil microbiota in a sustainable olive orchard. Soil Sci..

[50] Sofo A., Ciarfaglia A., Scopa A., Camele I., Curci M., Crecchio C., Xiloyannis C., Palese A.M. (2014). Soil microbial diversity and activity in a Mediterranean olive orchard using sustainable agricultural practices. Soil Use Manag..

[51] Montes-Borrego M., Metsis M., Landa B.B. (2014). Arbuscular mycorhizal fungi associated with the olive crop across the Andalusian landscape: Factors driving community differentiation. PLoS ONE.

[52] Landa B.B., Montes M., Aranda S., Soriano M.A., Gómez J.A., Navas J.A. (2014). Soil factors involved in the diversity and structure of soil bacterial communities in commercial organic olive orchards in southern Spain. Environ. Microbiol. Rep..

[53] Moreno B., Garcia-Rodriguez S., Cañizares R., Castro J., Benítez E. (2009). Rainfed olive farming in south-eastern Spain: Long-term effect of soil management on biological indicators of soil quality. Agric. Ecosyst. Environ..

[54] Caliz J., Montes-Borrego M., Triadó-Margarit X., Metsis M., Landa B.B., Casamayor E.O. (2015). Influence of edaphic, climatic, and agronomic factors on the composition and abundance of nitrifying microorganisms in the rhizosphere of commercial olive crops. PLoS ONE.

[55] De Graaff M.A., Classen A.T., Castro H.F., Schadt C.W. (2010). Labile soil carbon inputs mediate the soil microbial community composition and plant residue decomposition rates. New Phytol..

[56] Navarro-Noya Y.E., Gómez-Acata S., Montoya-Ciriaco N., Rojas-Valdez A., Suárez-Arriaga M.C., Valenzuela-Encinas C., Jiménez-Bueno N., Verhulst N., Govaerts B., Dendooven L. (2013). Relative impacts of tillage, residue management and crop-rotation on soil bacterial communities in a semi-arid agroecosystem. Soil Biol. Biochem..

[57] Ramirez-Villanueva D.A., Bello-López J.M., Navarro-Noya Y.E., Luna-Guido M., Verhulst N., Govaerts B., Dendooven L. (2015). Bacterial community structure in maize residue amended soil with contrasting management practices. Appl. Soil Ecol..

[58] Belmonte S.A., Celi l., Stahel R.J., Bonifacio E., Novello V., Zanini E., Steenwerth K.L. (2018). Effect of Long-Term Soil Management on the Mutual Interaction Among Soil Organic Matter, Microbial Activity and Aggregate Stability in a Vineyard. Pedosphere.

[59] Wortman S.E., Francis C., Bernards M.L., Drijber R.A., Lindquist J.L. (2012). Optimizing Cover Crop Benefits with Diverse Mixtures and an Optimizing Cover Crop Benefits with Diverse Mixtures and an Alternative Termination Method Alternative Termination Method. Agr. J..

[60] Hamel C., Vujanovic V., Jeannotte R., Nakano-Hylander A., St-Arnaud M. (2005). Negative feedback on a perennial crop: Fusarium crown and root rot of asparagus is related to changes in soil microbial community structure. Plant Soil.

[61] Mazzola M., Manici L.M. (2012). Apple Replant Disease: Role of Microbial Ecology in Cause and Control. Annu. Rev. Phytopathol..

[62] Bever J.D., Mangan S.A., Alexander H.M. (2015). Maintenance of Plant Species Diversity by Pathogens. Annu. Rev. Ecol. Evol. Syst..

[63] Garbeva P., van Veen J.A., van Elsas J.D. (2004). MICROBIAL DIVERSITY IN SOIL: Selection of Microbial Populations by Plant and Soil Type and Implications for Disease Suppressiveness. Annu. Rev. Phytopathol..

[64] Maron J.L., Marler M., Klironomos J.N., Cleveland C.C. (2011). Soil fungal pathogens and the relationship between plant diversity and productivity. Ecol. Lett..

[65] Fanin N., Hättenschwiler S., Fromin N. (2014). Litter fingerprint on microbial biomass, activity, and community structure in the underlying soil. Plant Soil.

[66] Civitello D.J., Cohen J., Fatima H., Halstead N.T., Liriano J., McMahon T.A., Ortega C.N., Sauer E.L., Sehgal T., Young S. (2015). Biodiversity inhibits parasites: Broad evidence for the dilution effect. Proc. Natl. Acad. Sci. USA.

[67] Palese A.M., Vignozzi N., Celano G., Agnelli A.E., Pagliai M., Xiloyannis C. (2014). Influence of soil management on soil physical characteristics and water storage in a mature rainfed olive orchard. Soil Tillage Res..

[68] Almagro M., Martinez-Mena M. (2014). Litter decomposition rates of green manure as affected by soil erosion, transport and deposition processes, and the implications for the soil carbon balance of a rainfed olive grove under a dry Mediterranean climate. Agric. Ecosyst. Environ..

[69] Parras-Alcántara L., Lozano-García B., Keesstra S., Cerdà A., Brevik E.C. (2016). Long-term effects of soil management on ecosystem services and soil loss estimation in olive grove top soils. Sci. Total Environ..

[70] Zuazo V.H.D., Pleguezuelo C.R.R., Martínez J.R.F., Raya A.M., Panadero L.A., Rodríguez B.C., Moll M.C.N. (2008). Benefits of plant strips for sustainable mountain agriculture. Agron. Sustain. Dev..

[71] Zuazo V.H.D., Pleguezuelo C.R.R., Panadero L.A., Raya A.M., Martínez J.R.F., Rodríguez B.C. (2009). Soil Conservation Measures in Rainfed Olive Orchards in South-Eastern Spain: Impacts of Plant Strips on Soil Water Dynamics. Pedosphere.

[72] Fess T.L., Benedito V.A. (2018). Organic versus Conventional Cropping Sustainability: A Comparative System Analysis. Sustainability.

[73] TerAvest D., Smith J.L., Carpenter-Boggs L., Hoagland L., Granatstein D., Reganold J.P. (2010). Influence of orchard floor management and compost application timing on nitrogen partitioning in apple trees. HortScience.

[74] Dabney S.M., Meisinger J.J., Schomberg H.H., Liebig M.A., Kaspar T.C., Delgado J.A., Mitchell J., Reeves D.W., Delgado J.A., Follett R.F. (2010). Using cover crops and cropping systems for nitrogen management. Advances in Nitrogen Management for Water Quality.

[75] Peix A., Ramírez-Bahena M.H., Velázquez E., Bedmar E.J. (2015). Bacterial Associations with Legumes. CRC. Crit. Rev. Plant Sci..

[76] Gaby J.C., Buckley D.H. (2014). A comprehensive aligned nifH gene database: A multipurpose tool for studies of nitrogen-fixing bacteria. Database.

[77] Reed S.C., Cleveland C.C., Townsend A.R. (2011). Functional Ecology of Free-Living Nitrogen Fixation: A Contemporary Perspective. Annu. Rev. Ecol. Evol. Syst..

[78] Wei W., Isobe K., Nishizawa T., Zhu L., Shiratori Y., Ohte N., Koba K., Otsuka S., Senoo K. (2015). Higher diversity and abundance of denitrifying microorganisms in environments than considered previously. ISME J..

[79] Condron L., Stark C., O’Callaghan M., Clinton P., Huang Z. (2010). The Role of Microbial Communities in the Formation and Decomposition of Soil Organic Matter. Soil Microbiology and Sustainable Crop Production.

[80] Smercina D.N., Evans S.E., Friesen M.L., Tiemann L.K. (2019). To fix or not to fix: Controls on free-living nitrogen fixation in the rhizosphere. Appl. Environ. Microbiol..

[81] Vitousek P.M., Cassman K., Cleveland C., Crews T., Field C.B., Grimm N.B., Howarth R.W., Marino R., Martinelli L., Rastetter E.B. (2002). Towards an ecological understanding of biological nitrogen fixation. Biogeochemistry.

[82] Morales S.E., Cosart T., Holben W.E. (2010). Bacterial gene abundances as indicators of greenhouse gas emission in soils. ISME J..

[83] Hink L., Gubry-Rangin C., Nicol G.W., Prosser J.I. (2018). The consequences of niche and physiological differentiation of archaeal and bacterial ammonia oxidisers for nitrous oxide emissions. ISME J..

[84] Gubry-Rangin C., Hai B., Quince C., Engel M., Thomson B.C., James P., Schloter M., Griffiths R.I., Prosser J.I., Nicol G.W. (2011). Niche specialization of terrestrial archaeal ammonia oxidizers. Proc. Natl. Acad. Sci. USA.

[85] Nicol G.W., Leininger S., Schleper C., Prosser J.I. (2008). The influence of soil pH on the diversity, abundance and transcriptional activity of ammonia oxidizing archaea and bacteria. Environ. Microbiol..

[86] Prosser J.I., Nicol G.W. (2012). Archaeal and bacterial ammonia-oxidisers in soil: The quest for niche specialisation and differentiation. Trends Microbiol..

[87] Hai B., Diallo N.H., Sall S., Haesler F., Schauss K., Bonzi M., Assigbetse K., Chotte J.L., Munch J.C., Schloter M. (2009). Quantification of key genes steering the microbial nitrogen cycle in the rhizosphere of sorghum cultivars in tropical agroecosystems. Appl. Environ. Microbiol..

[88] Wang Y., Zhu G., Song L., Wang S., Yin C. (2014). Manure fertilization alters the population of ammonia-oxidizing bacteria rather than ammonia-oxidizing archaea in a paddy soil. J. Basic Microbiol..

[89] Tu Q., He Z., Wu L., Xue K., Xie G., Chain P., Reich P.B., Hobbie S.E., Zhou J. (2017). Metagenomic reconstruction of nitrogen cycling pathways in a CO2-enriched grassland ecosystem. Soil Biol. Biochem..

[90] Zumft W.G. (1997). Cell biology and molecular basis of denitrification. Microbiol. Mol. Biol. Rev..

[91] Tiedje J.M., Sexstone A.J., Myrold D.D., Robinson J.A. (1983). Denitrification: Ecological niches, competition and survival. Antonie Van Leeuwenhoek.

[92] Richardson A.E., Hocking P.J., Simpson R.J., George T.S. (2009). Plant mechanisms to optimise access to soil phosphorus. Proceedings of the Crop and Pasture Science.

[93] Wei L., Chen C., Xu Z. (2010). Citric acid enhances the mobilization of organic phosphorus in subtropical and tropical forest soils. Biol. Fertil. Soils.

[94] Spohn M., Kuzyakov Y. (2013). Phosphorus mineralization can be driven by microbial need for carbon. Soil Biol. Biochem..

[95] Acuña J.J., Durán P., Lagos L.M., Ogram A., de la Luz Mora M., Jorquera M.A. (2016). Bacterial alkaline phosphomonoesterase in the rhizospheres of plants grown in Chilean extreme environments. Biol. Fertil. Soils.

[96] Sakurai M., Wasaki J., Tomizawa Y., Shinano T., Osaki M. (2008). Analysis of bacterial communities on alkaline phosphatase genes in soil supplied with organic matter. Soil Sci. Plant. Nutr..

[97] Zappa S., Rolland J.L., Flament D., Gueguen Y., Boudrant J., Dietrich J. (2001). Characterization of a Highly Thermostable Alkaline Phosphatase from the Euryarchaeon Pyrococcus abyssi. Appl. Environ. Microbiol..

[98] Sebastian M., Ammerman J.W. (2009). The alkaline phosphatase PhoX is more widely distributed in marine bacteria than the classical PhoA. ISME J..

[99] Magnusson O.T., Toyama H., Saeki M., Rojas A., Reed J.C., Liddington R.C., Klinman J.P., Schwarzenbacher R. (2004). Quinone biogenesis: Structure and mechanism of PqqC, the final catalyst in the production of pyrroloquinoline quinone. Proc. Natl. Acad. Sci. USA.

[100] Greiner R., Konietzny U., Blackburn D.M., Jorquera M.A. (2013). Production of partially phosphorylated myo-inositol phosphates using phytases immobilised on magnetic nanoparticles. Bioresour. Technol..

[101] Alori E.T., Glick B.R., Babalola O.O. (2017). Microbial phosphorus solubilization and its potential for use in sustainable agriculture. Front. Microbiol..

[102] Koide R.T., Kabir Z. (2000). Extraradical hyphae of the mycorrhizal fungus Glomus intraradices can hydrolyse organic phosphate. New Phytol..

[103] Malmstrom R.R., Eloe-Fadrosh E.A. (2019). Advancing Genome-Resolved Metagenomics beyond the Shotgun. mSystems.

[104] Douglas G.M., Maffei V.J., Zaneveld J., Yurgel S.N., Brown J.R., Taylor C.M., Huttenhower C., Langille M.G.I. (2019). PICRUSt2: An improved and extensible approach for metagenome inference. bioRxiv.

[105] Wemheuer F., Taylor J.A., Daniel R., Johnston E., Meinicke P., Thomas T., Wemheuer B. (2018). Tax4Fun2: A R-based tool for the rapid prediction of habitat-specific functional profiles and functional redundancy based on 16S rRNA gene marker gene sequences. bioRxiv.

[106] Louca S., Parfrey L.W., Doebeli M. (2016). Decoupling function and taxonomy in the global ocean microbiome. Science.

[107] Barberán A., Bates S.T., Casamayor E.O., Fierer N. (2012). Using network analysis to explore co-occurrence patterns in soil microbial communities. ISME J..

[108] Röttjers L., Faust K. (2018). From hairballs to hypotheses–biological insights from microbial networks. FEMS Microbiol. Rev..

[109] Yang P., Yu S., Cheng L., Ning K. (2019). Meta-network: Optimized species-species network analysis for microbial communities. BMC Genomics.

[110] Berry D., Widder S. (2014). Deciphering microbial interactions and detecting keystone species with co-occurrence networks. Front. Microbiol..

[111] Herren C.M., McMahon K.D. (2018). Keystone taxa predict compositional change in microbial communities. Environ. Microbiol..

[112] Bünemann E.K., Bongiorno G., Bai Z., Creamer R.E., De Deyn G., de Goede R., Fleskens L., Geissen V., Kuyper T.W., Mäder P. (2018). Soil quality – A critical review. Soil Biol. Biochem..

[113] Schloter M., Nannipieri P., Sørensen S.J., van Elsas J.D. (2018). Microbial indicators for soil quality. Biol. Fertil. Soils.

[114] Tourna M., Stieglmeier M., Spang A., Könneke M., Schintlmeister A., Urich T., Engel M., Schloter M., Wagner M., Richter A. (2011). Nitrososphaera viennensis, an ammonia oxidizing archaeon from soil. Proc. Natl. Acad. Sci. USA.

[115] Zhalnina K., Dörr de Quadros P., Camargo F.A.O., Triplett E.W. (2012). Drivers of archaeal ammonia-oxidizing communities in soil. Front. Microbiol..

[116] Zhalnina K., de Quadros P.D., Gano K.A., Davis-Richardson A., Fagen J.R., Brown C.T., Giongo A., Drew J.C., Sayavedra-Soto L.A., Arp D.J. (2013). Ca. Nitrososphaera and Bradyrhizobium are inversely correlated and related to agricultural practices in long-term field experiments. Front. Microbiol..

[117] Wang B., Zheng Y., Huang R., Zhou X., Wang D., He Y., Jia Z. (2014). Active ammonia oxidizers in an acidic soil are phylogenetically closely related to neutrophilic archaeon. Appl. Environ. Microbiol..

[118] Frac M., Hannula S.E., Belka M., Jȩdryczka M. (2018). Fungal biodiversity and their role in soil health. Front. Microbiol..

